# Mechanical Characterization and Modeling of Glass Fiber-Reinforced Polyamide Built by Additive Manufacturing

**DOI:** 10.3390/ma18040745

**Published:** 2025-02-08

**Authors:** Massimiliano Avalle, Mattia Frascio

**Affiliations:** Dipartimento di Ingegneria Meccanica, Energetica, Gestionale e dei Trasporti, Università di Genova, 16145 Genova, Italy; mattia.frascio@unige.it

**Keywords:** additive manufacturing, composite materials, classical lamination theory, strength criteria for composites, long-fiber-reinforced polyamide

## Abstract

Additive manufacturing (AM) is an emerging technology with the greatest potential impact on many engineering applications. Among the AM technologies, material extrusion is particularly interesting for plastic components due to its versatility and cost-effectiveness. There is, however, a limited knowledge of design methods to predict the mechanical strength of parts built by material extrusion. The materials are polymers, sometimes also reinforced, and deposited in layers like in laminated composites. Therefore, the mechanical behavior and strength can be characterized and modeled with methods already known for composite materials. Such tools are the classical lamination theory (CLT) and the failure criteria for composites. This paper addresses an analysis of a composite material made of long-fiber glass in a polyamide matrix built by additive manufacturing; in this relatively new technique, a continuous fiber is inserted between layers of polyamide deposited from a wire with a fused filament fabrication (FFF) 3D printer. The mechanical behavior was studied from tensile tests that were carried out to demonstrate the feasibility of modeling with the mentioned tools, and the material properties for predicting the stiffness and strength of components built with that technique were identified. The results show that the classical models for the mechanical behavior of composite materials are well-suited for this material to predict the influence of the main building parameters.

## 1. Introduction

The additive manufacturing (AM) techniques are gaining an ever-growing attention in many engineering applications due to their advantages in terms of endless possibilities to generate new designs impossible with other technologies and—more recently—even with advantages in terms of reduction in cost and material waste [1]. On the other hand, there are still many issues preventing a wide-scale adoption of AM technology related to cost and productivity [2]. These considerations often restrict the industrial use of AM to small-scale production, such as in aerospace and luxury cars [3]. Among the numerous AM techniques for plastics, the material extrusion (ME, ASTM F2792-12) is particularly interesting [4]; mostly known as fused filament fabrication (FFF) or with the trademark name Fused Deposition Modeling™ (FDM, Stratasys, Inc.), it is based on the deposition of a fused wire of a polymeric material on a plate and then, layer by layer, on the construction of the component by progressive addition of material [5]. In addition to the low cost of this technology, a main advantage of FFF lies in the way the material is used, that is, by melting a thermoplastic, which allows extensive choices in terms of basic raw materials [6], charged composites [7], as well as multifunctional [8] and sensorial metamaterials [9,10]. Of course, FFF is not without limitations: first of all, there is the weak mechanical strength often caused by poor adhesion between the layers, and then there is the poor surface finish and difficulties in precisely controlling the size, tolerances, and porosity [11,12]. The ability to 3D print long-fiber composites is a relatively recent extension with a potentially great impact on the applications of FFF [13,14]. Among the main reinforcements available for FFF processing, there are glass fibers [15], carbon fibers [16,17], and aramid fibers [18]. Glass fiber reinforcement is a good compromise between cost and performance and is interesting for biomedical applications, while carbon fibers give the best mechanical strength [19]; aramid fibers are known for their toughness, which makes them interesting for applications in sport and protection equipment [20].

The properties of components obtained by AM depend on a lot of different parameters, obviously the first being the nature of the material [6], others related to the processing parameters [21], and their combinations. Many studies were devoted, in fact, to examining the process parameters for several well-known plastics, such as the widely spread PLA [22,23,24], ABS [25,26,27,28], PC [28], and ASA [29]. Moreover, the adhesion to the plate and between the layers is critical for the quality of FFF parts; this has been studied, for example, for ABS [30] and PC [30], also considering the effect of temperature.

Processing parameters and their influence are many, and several studies have been carried out; among the others, the main parameters of influence include the infill density and infill patterns [23,29], the layer thickness [24,26,29], and the layer gap [25]. Many papers consider the various parameters for optimization; a review is given in [31].

The infill pattern plays an important role, and even with a simple linear pattern, it has been demonstrated that the way the filament is deposited can have a significant influence on the stiffness and strength of components built by FFF. An analysis of the influence of the orientation of the fused wire was carried out by many authors, among them Wu et al. with PEEK and ABS [26]; Somireddy et al. [32], Ahn et al. [25], Alaimo et al. [33], and Croccolo et al. for ABS [27]; Rajpurohit et al. [34], Lanzotti et al. [22], Dai et al. [35], and Yilmaz et al. [36] with PLA; and Avalle et al. with PETG and PA12 [37]. Many other influencing factors are important for the performances of FFF composites, including post-processing and surface treatments, and are covered in the recent literature, such as [38].

Therefore, since a typical infill is by stacked layers of parallel filaments, it is natural to compare this type of structured arrangement equivalent to the stack of a classical laminated composite. The layered structure built by FFF can therefore be studied with methods used for composites for decades that form the so-called classical lamination theory (CLT). The CLT is a homogenization method developed in the 20th century [39,40,41] to predict the mechanical behavior of layered composites made by assembling layers of a reinforced polymeric matrix with various stacking, thicknesses, and fiber orientations. This idea proposed some years ago by Casavola et al. [42], and has been used to characterize the behavior of layered FFF specimens or components by several authors and with different materials and different stacking. Somireddy et al. [32] used the technique to study ABS cross-ply stacking at 0°, 45°, and 90° angles, and similarly with PLA in [34,36]; Musenich et al. [10] considered the analysis of a conductive filament obtained by charging PLA with carbon nano-particles; Alaimo et al. [33] considered more orientation but aligned filaments in all layers. Nasirov et al. [43] compared the results from the CLT with a finite element model, finding a good correlation between the models. Simulation is an alternative method [44] with the advantages and disadvantages of numerical models. It is worth remembering that the CLT is often included in 2D models of most engineering structures modeled by shell elements.

The analysis of the strength of layered specimens built by FFF can be carried out by using the failure criteria of composite materials. This study is more complex since the failure of composites relies on several failure criteria proposed since the mid-20th century. In [37], Avalle et al. used the Tsai–Hill [45,46] and Tsai–Wu [47] criteria to model the failure of PETG and PA12. Musenich et al. [10] similarly considered the PLA-charged material with the Tsai–Wu failure criterion. Both found a good correlation with the experimental results.

In this work, the results from experimental tests on layered specimens made of a fiber-reinforced polyamide are reported and analyzed. The specimens were built layered by FFF using a symmetric angle-ply configuration with variable orientations of the fibers. The experimental results clearly show the strong influence of fiber orientation on both stiffness and strength. The adoption of the CLT and of different failure criteria shows the applicability of the method to forecast the mechanical properties of components built by FFF. The mechanical properties in terms of layer stiffness and strength could be identified for this purpose.

## 2. Materials and Methods

### 2.1. Mechanical Behavior of a Lamina and Laminate

Most composite materials are made of a stack of unidirectional or woven laminae. The mechanical behavior of a single lamina is typically orthotropic along the fiber direction. The relation between stress and strain in the *x–y* lamina plane is described by the following well-known relation [39,40,41]:

(1) $$\left\{\begin{array}{c} \varepsilon_{xx}\\ \varepsilon_{yy}\\ \gamma_{xy} \end{array}\right\}=\left[\begin{array}{ccc} S_{xx} & S_{xy} & 0\\ S_{xy} & S_{yy} & 0\\ 0 & 0 & S_{ss} \end{array}\right]\left\{\begin{array}{c} \sigma_{xx}\\ \sigma_{yy}\\ \sigma_{xy} \end{array}\right\}$$

where the terms *Q_ij_* are related to the material constants by the following classical relation:

(2) $$\left\{\begin{array}{c} \begin{array}{c} S_{xx}=\frac{1}{E_{xx}}\\ S_{xy}=-\frac{\nu_{xy}}{E_{xx}}=-\frac{\nu_{yx}}{E_{yy}}\\ S_{yy}=\frac{1}{E_{yy}} \end{array}\\ S_{ss}=\frac{1}{G_{xy}} \end{array}\right.$$

With *E_xx_*, *E_yy_*, *ν_xy_,* and *G_xy_* being the elastic modulus along the fiber direction, the elastic modulus in the transverse direction, the coefficient of transverse contraction, and the tangential modulus, respectively.

The inverse relation is given in Equation (3) as follows:

(3) $$\left\{\begin{array}{c} \sigma_{xx}\\ \sigma_{yy}\\ \tau_{xy} \end{array}\right\}=\left[\begin{array}{ccc} Q_{xx} & Q_{xy} & 0\\ Q_{xy} & Q_{yy} & 0\\ 0 & 0 & Q_{ss} \end{array}\right]\left\{\begin{array}{c} \varepsilon_{xx}\\ \varepsilon_{yy}\\ \gamma_{xy} \end{array}\right\}$$

Since the laminae are not generally stacked and aligned along a single direction, their behavior in a generic reference system, common to all the stacked laminae, is anisotropic. By means of simple trigonometric transformations, the stress–strain relations in a generic common direction 1–2 are as follows:

(4) $$\left\{\begin{array}{c} \sigma_{1}\\ \sigma_{2}\\ \tau_{12} \end{array}\right\}=\left[\begin{array}{ccc} Q_{11} & Q_{12} & Q_{16}\\ Q_{12} & Q_{22} & Q_{26}\\ Q_{16} & Q_{26} & Q_{66} \end{array}\right]\left\{\begin{array}{c} \varepsilon_{1}\\ \varepsilon_{2}\\ \gamma_{12} \end{array}\right\}$$

The mechanical behavior of a laminate is, in the simplest approach, obtained from the Kirchoff–Love hypothesis; restricting to the in-plane membrane behavior, the relations between the laminate stress and the in-plane strain are as follows:

(5) $$\left\{\begin{array}{c} \sigma_{1}\\ \sigma_{2}\\ \tau_{12} \end{array}\right\}=\frac{1}{t}\left[\begin{array}{ccc} A_{11} & A_{12} & A_{16}\\ A_{12} & A_{22} & A_{26}\\ A_{16} & A_{26} & A_{66} \end{array}\right]\left\{\begin{array}{c} \varepsilon_{1}\\ \varepsilon_{2}\\ \gamma_{12} \end{array}\right\}$$

with *t* being the thickness of the laminate, the sum of the individual thicknesses of the stacked layers. The laminate’s constitutive equations are obtained from the summation of the contribution of each single lamina to the total stiffness:

(6) $$A_{ij}=\sum_{k=1}^{n}\left(z_{k}-z_{k-1}\right)Q_{ij,k}$$

With *n* being the number of layers, *z_k_* and *z_k_*_−1_ being the top and bottom layer distance from the midsection of the laminate of the *k*-th layer, and in *Q_ij_*_,*k*_, *ij* is the stiffness coefficient of the *k*-th layer. In the case of a symmetric laminate, the terms *A*_16_ and *A*_26_ are zero due to the cancellation of the contributions from the symmetric layers.

When subjecting a specimen to simple tension, the stress–strain relations allow for computing the apparent elastic modulus of the laminate. In turn, since the laminate mechanical constants derive from the contribution of the stacked layers, it would be possible to identify the layers’ mechanical properties, as from Equation (2).

When subjecting a laminate specimen to pure tensile and avoiding spurious in-plane shear deformation with symmetric stacking, two cases can occur: In the case of uniaxial deformation (UD), the global laminate stress–strain relation is as follows (directly derived from Equation (5)):

(7) $$\left\{\begin{array}{c} \sigma_{1}\\ \sigma_{2}\\ \tau_{12} \end{array}\right\}=\frac{1}{t}\left[\begin{array}{ccc} A_{11} & A_{12} & A_{16}\\ A_{12} & A_{22} & A_{26}\\ A_{16} & A_{26} & A_{66} \end{array}\right]\left\{\begin{array}{c} \varepsilon_{1}\\ 0\\ 0 \end{array}\right\}$$

The apparent elastic modulus will be simply

(8) $$\sigma_{1}=\frac{1}{t}A_{11}\varepsilon_{1}\;\;\to \;\;E_{app,UD}=\frac{A_{11}}{t}\;$$

In the case of uniaxial stress (US) instead, the stress–strain relation is

(9) $$\left\{\begin{array}{c} \sigma_{1}\\ 0\\ 0 \end{array}\right\}=\frac{1}{t}\left[\begin{array}{ccc} A_{11} & A_{12} & A_{16}\\ A_{12} & A_{22} & A_{26}\\ A_{16} & A_{26} & A_{66} \end{array}\right]\left\{\begin{array}{c} \varepsilon_{1}\\ \varepsilon_{2}\\ \gamma_{12} \end{array}\right\}$$

The apparent elastic modulus will be simply

(10) $$\sigma_{1}=\frac{1}{t}\left(A_{11}-\frac{A_{12}^{2}}{A_{22}}\right)\varepsilon_{1}\;\;\to \;\;E_{app,US}=\frac{1}{t}\left(A_{11}-\frac{A_{12}^{2}}{A_{22}}\right)\;$$

Standard tensile tests according to ISO 527-1 [48] or ASTM D 638 [49] are better considered in a state of uniaxial stress; therefore, Equation (10) will be used to show that a layered structure obtained by material extrusion with FFF behaves like a laminated composite and, consequently, to identify the elastic properties of the layers. Identification will be carried out by testing symmetric angle-ply specimens with different orientations of the layer and with a non-linear regression analysis of the apparent modulus with variable moduli, as in Equation (2).

### 2.2. Mechanical Strength of a Lamina and Laminate

Failure of polymeric and composite materials is way more complex than in metals due to both their non-linear behavior and anisotropy. Although the anisotropic behavior of metals is sometimes recognized, the effects of anisotropy are considered negligible, thus bringing relatively simple failure criteria such as the well-known one by Von Mises. An extension of the Von Mises criterion is due to Hill [50], which is also the basis for some failure criteria for composites. First of all, the Tsai–Hill [45,46] criterion is a validated method, used for decades, to model the failure of composites in multiaxial loading. The Tsai–Hill criterion states that failure does not occur in a layer when the following condition is met:

(11) $${\left(\frac{\sigma_{xx}}{X}\right)}^{2}-\frac{\sigma_{xx}\sigma_{yy}}{X^{2}}+{\left(\frac{\sigma_{yy}}{Y}\right)}^{2}+{\left(\frac{\sigma_{xy}}{S}\right)}^{2}\leq 1\;$$

where *X*, *Y*, and *S* are the strength of each layer along the fiber direction *x*, in the transverse direction *y*, and in shear. The Tsai–Hill criterion has several limitations; first, there is no distinction between tension and compression.

In the case of uniaxial loading in the 1 direction, the uniaxial stress *σ*_1_ should be limited to

(12) $$\frac{\sigma_{1}^{2}\cos^{4}\theta }{X^{2}}-\frac{\sigma_{1}^{2}\cos^{2}\theta \sin^{2}\theta }{X^{2}}+\frac{\sigma_{1}^{2}\sin^{4}\theta }{Y^{2}}+\frac{\sigma_{1}^{2}\cos^{2}\theta \sin^{2}\theta }{S^{2}}\leq 1\;$$

which can be simplified as

(13) $$\sigma_{1}^{2}\left(\frac{\cos^{4}\theta }{X^{2}}-\frac{\cos^{2}\theta \sin^{2}\theta }{X^{2}}+\frac{\sin^{4}\theta }{Y^{2}}+\frac{\cos^{2}\theta \sin^{2}\theta }{S^{2}}\right)\leq 1\;$$

As for the elastic properties, the strength coefficients can be identified with a non-linear regression analysis of the uniaxial strength of a layered symmetric angle-ply specimen with different orientations:

(14) $$\sigma_{1}\leq \frac{1}{\sqrt{\frac{\cos^{4}\theta }{X^{2}}-\frac{\cos^{2}\theta \sin^{2}\theta }{X^{2}}+\frac{\sin^{4}\theta }{Y^{2}}+\frac{\cos^{2}\theta \sin^{2}\theta }{S^{2}}}}=S_{1}\;$$

Further development of this criterion led to the Tsai–Wu criterion [47,51], which allows for distinguishing between failure in tension and in compression:

(15) $$\frac{\sigma_{x}^{2}}{X_{t}X_{c}}-{2F}_{xy}^{*}\frac{\sigma_{x}\sigma_{y}}{\sqrt{X_{t}X_{c}Y_{t}Y_{c}}}+\frac{\sigma_{y}^{2}}{Y_{t}Y_{c}}+{\left(\frac{\sigma_{xy}}{S}\right)}^{2}+\frac{\left(X_{c}-X_{t}\right)\sigma_{x}}{X_{t}X_{c}}+\frac{\left(Y_{c}-Y_{t}\right)\sigma_{y}}{Y_{t}Y_{c}}\leq 1\;$$

where *X_t_* and *X_c_* are the strength in tension and compression, respectively, of each layer along the fiber direction, *Y_t_* and *Y_c_* are the strength in tension and compression, respectively, along the fiber direction, S is the shear strength, and $F_{xy}^{*}$ is a correlation coefficient. The Tsai–Wu criterion is more suitable for composite materials, which are known to have different strengths in tension rather than in compression.

In the case of uniaxial loading in the 1 direction, the condition is then

(16) $$\frac{\sigma_{1}^{2}\cos^{4}\theta }{X_{t}X_{c}}-{2F}_{xy}^{*}\frac{\sigma_{1}^{2}\cos^{2}\theta \sin^{2}\theta }{\sqrt{X_{t}X_{c}Y_{t}Y_{c}}}+\frac{\sigma_{1}^{2}\sin^{4}\theta }{Y_{t}Y_{c}}+\frac{\sigma_{1}^{2}\cos^{2}\theta \sin^{2}\theta }{S^{2}}+\frac{\left(X_{c}-X_{t}\right)\sigma_{1}^{2}\cos^{4}\theta }{X_{t}X_{c}}+\frac{\left(Y_{c}-Y_{t}\right)\sigma_{1}^{2}\sin^{2}\theta }{Y_{t}Y_{c}}\leq 1\;$$

The strength *S*_1_ of each layer subject to unidirectional loading is then

(17) $$\sigma_{1}\leq \frac{1}{\sqrt{\frac{\cos^{4}\theta }{X_{t}X_{c}}-{2F}_{xy}^{*}\frac{\cos^{2}\theta \sin^{2}\theta }{\sqrt{X_{t}X_{c}Y_{t}Y_{c}}}+\frac{\sin^{4}\theta }{Y_{t}Y_{c}}+\frac{\cos^{2}\theta \sin^{2}\theta }{S^{2}}+\frac{\left(X_{c}-X_{t}\right)\cos^{4}\theta }{X_{t}X_{c}}+\frac{\left(Y_{c}-Y_{t}\right)\sin^{2}\theta }{Y_{t}Y_{c}}}}=S_{1}\;$$

However, both these criteria do not allow for distinguishing between different failure modes; it is well known that in composite materials, failure can happen in several modes due to the heterogeneity of the components. Some typical failure modes are fiber failure in tension along the fiber direction, matrix cracking along the transverse direction, fiber buckling in compression along the fiber direction, shear failure of the matrix, and delamination. To take into account these differences, several criteria have been proposed; most propose a combination between the failure mode of the fiber and of the matrix. One of the first criterion is by Hashin [52,53] and is stated as

(18) $$\left\{\begin{array}{cc} {\left(\frac{\sigma_{xy}}{S}\right)}^{2}+{\left(\frac{\sigma_{y}}{Y_{T}}\right)}^{2}\leq 1 & matrix\;failure\\ {\left(\frac{\sigma_{x}}{X_{t}}\right)}^{2}+{\left(\frac{\sigma_{xy}}{S}\right)}^{2}\leq 1 & fiber\;failure \end{array}\right.\;$$

In unidirectional loading, the criterion becomes

(19) $$\left\{\begin{array}{cc} {{\left(\frac{\sigma_{1}\sin^{2}\theta }{Y_{T}}\right)}^{2}+\left(\frac{\sigma_{1}\sin\theta \cos\theta }{S_{12}}\right)}^{2}\leq 1 & matrix\;failure\\ {\left(\frac{\sigma_{1}\cos^{2}\theta }{X_{T}}\right)}^{2}+{\left(\frac{\sigma_{1}\sin\theta \cos\theta }{S_{12}}\right)}^{2}\leq 1 & fiber\;failure \end{array}\right.\;$$

which allows for identifying the layer strength in the unidirectional loading of each layer as

(20) $$\left\{\begin{array}{cc} \sigma_{1}\leq \frac{Y_{t}S}{\sqrt{S^{2}\sin^{4}\theta +Y_{T}^{2}\sin^{2}\theta \cos^{2}\theta }}=S_{1,MF} & matrix\;failure\\ \sigma_{1}\leq \frac{X_{t}S}{\sqrt{S^{2}\cos^{4}\theta +X_{T}^{2}\sin^{2}\theta \cos^{2}\theta }}=S_{1,FF} & fiber\;failure \end{array}\right.\;$$

Further development of this criterion is by Christensen [54], who introduces differentiation between failure in tension and in compression:

(21) $$\left\{\begin{array}{cc} \left(\frac{1}{Y_{t}}+\frac{1}{Y_{c}}\right)\sigma_{y}+\frac{1}{Y_{t}Y_{c}}\sigma_{y}^{2}+\frac{\sigma_{xy}^{2}}{S^{2}}\leq 1 & matrix\;failure\\ \left(\frac{1}{X_{t}}+\frac{1}{X_{c}}\right)\sigma_{x}+\frac{1}{X_{t}X_{c}}\sigma_{x}^{2}\leq 1 & fiber\;failure \end{array}\right.\;$$

In unidirectional loading, the criterion becomes

(22) $$\left\{\begin{array}{cc} \left(\frac{1}{Y_{t}}+\frac{1}{Y_{c}}\right)\sigma_{1}\sin^{2}\theta +\frac{1}{Y_{t}Y_{c}}\sigma_{1}^{2}\sin^{4}\theta +\frac{\sigma_{1}^{2}\sin^{2}\theta \cos^{2}\theta }{S^{2}}\leq 1 & matrix\;failure\\ \left(\frac{1}{X_{t}}+\frac{1}{X_{c}}\right)\sigma_{1}\cos^{2}\theta +\frac{1}{X_{t}X_{c}}\sigma_{1}^{2}\cos^{4}\theta \leq 1 & fiber\;failure \end{array}\right.\;$$

A similar approach was used to define several other criteria even more complex in terms of governing parameters. Among those criteria, the simpler LaRC05 model [55,56] is worth mentioning:

(23) $$\left\{\begin{array}{cc} {\left(\frac{\sigma_{xy}}{S+\eta_{T}\sigma_{y}}\right)}^{2}+{\left(\frac{\sigma_{y}}{Y_{T}}\right)}^{2}\leq 1 & matrix\;failure\\ \frac{\sigma_{x}}{X_{t}}\leq 1 & fiber\;failure \end{array}\right.\;$$

In unidirectional loading, the criterion becomes

(24) $$\left\{\begin{array}{cc} {\left(\frac{\sigma_{1}\sin\theta \cos\theta }{S_{12}+\eta_{T}\sigma_{1}\sin^{2}\theta }\right)}^{2}+{\left(\frac{\sigma_{1}\sin^{2}\theta }{Y_{T}}\right)}^{2}\leq 1 & matrix\;failure\\ \left(\frac{1}{X_{t}}\right)\sigma_{1}\cos^{2}\theta \leq 1 & fiber\;failure \end{array}\right.\;$$

### 2.3. Manufacturing of Layered Composites by AM

Experimental results to apply the proposed models and identify the material properties were obtained from tensile tests on layered composites built by AM. The tensile test specimens were built with a Mark Two™ (MarkForged^®^, Waltham, MA, USA) printer able to deposit layers of continuous fibers of various types (glass, carbon, aramid) in a polyamide matrix. The deposition of the polymeric matrix and of the fiber is possible thanks to the use of two extruders, one for the matrix and the second for the fiber, and an automated blade to cut the fiber at the end of each composite layer.

**Figure 1:**
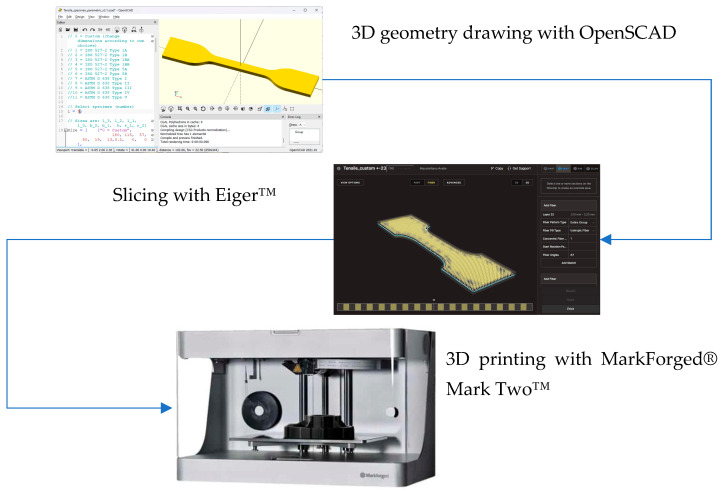
Specimen generation and 3D printing process.

The specimens were prepared for printing by using the dedicated proprietary slicing software Eiger™ (https://www.eiger.io/, accessed on 1 February 2025). The slicer starts by importing the geometry saved in an STL file, then it allows for defining the layer sequence. In particular, it allows for defining the number and sequence of composite layers and, for each layer, for defining the orientation of the fiber (at least for the basic pattern of fiber parallel and aligned in a single direction). Figure 1 aims to describe the preparation process of the samples.

**Figure 2:**
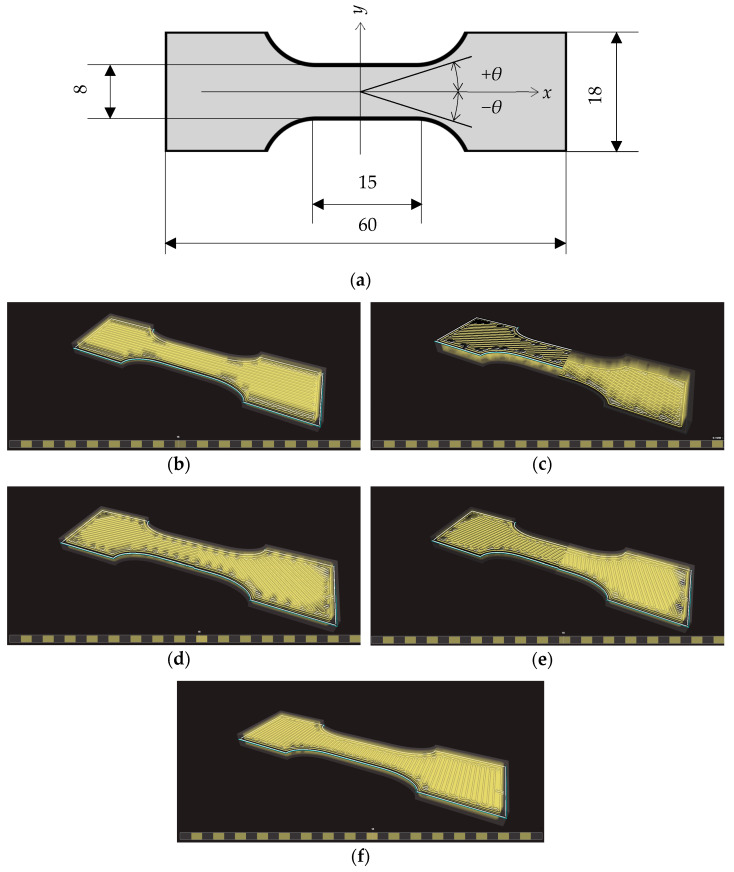
Geometry and layering scheme of the used specimen: (**a**) plane top section of the specimen, thickness 3.2 mm; (**b**) sample with fibers aligned at 0° orientation, image from Eiger™ slicing software; (**c**) fibers at ±23°; (**d**) fibers at ±45°; (**e**) fibers at ±67°; (**f**) fibers at 90°.

The STL file containing the geometry of the specimens was created with an automated script in OpenSCAD (https://www.openscad.org, accessed on 1 February 2025), a free software for creating solid CA models with a scripting language. The script allows for very quickly, efficiently, and parametrically generating the geometry of tensile specimens. In the current work, a standard geometry as in the ISO 527-1 or ASTM D 638 was not used due to restrictions related to the used printer. The classical larger specimens (like ISO 527-2, types 1A or 1B, [57]) are too bulky, expensive, and time-consuming, whereas with the smaller specimens (such as ISO 527-2 5A or 5B), it is impossible to obtain a deposition of the fiber compatible with the objectives of the current research (there are technical limitations due to the deposition of the fiber into smaller spaces, for example, due to the curvature of the filament, as is evident from Figure 2).

All the samples were 3D printed by alternating layers of continuous glass fiber (from MarkForged^®^) with layers of the polyamide-reinforced Onyx™ (also from MarkForged^®^; reinforcement is made of carbon microfibers). Basic properties of the two materials are shown in Table 1. Recently, some papers were published about fiber-reinforced Onyx. In [58,59], carbon-fiber specimens and components were studied; in [20], Kevlar-reinforced Onyx samples were characterized; in [60], Yun et al. examined the failure of fiberglass-reinforced Onyx, showing in particular the influence of the number of layers on the strength in tension.

The fiber deposition was carried out to obtain a symmetric, balanced, angle-ply structure according to a [±*θ*]_4s_ layering scheme, *θ* being the angle between the longitudinal axial direction of the specimen and the fiber direction. The angle *θ* = 0 (Figure 2b) corresponds to the fibers aligned to the longitudinal specimen axis, whereas for *θ* = 90°, the fibers are perpendicular to that axis (Figure 2f). Symmetry was chosen to avoid unwanted coupling of in-plane and bending behaviors; the balanced angle-ply arrangement was chosen to obtain a globally orthotropic structure to avoid coupling of tension with shear behavior. The number of layers was the minimum to obtain a sufficiently homogenized structure.

## 3. Results

### 3.1. Experimental Plan

The aim of the current work was to show that the classical lamination theory and the failure criteria used for composite materials can be used to describe the mechanical behavior and strength of layered components obtained by filament deposition. Additionally, these theories can be used to identify the elastic properties of the layers and their strength. These theories and data could be used for the design of components made by filament deposition.

To this aim, samples according to Figure 2 were manufactured with the Mark Two printer with a number of different orientations as shown in Table 2.

Note that [±*θ*]_4s_ correspond to the following sequence (Figure 2b): [+*θ*/−*θ*/+*θ*/−*θ*/+*θ*/−*θ*/+*θ*/−*θ*/−*θ*/+*θ*/−*θ*/+*θ*/−*θ*/+*θ*/−*θ*/+*θ*]. The cases when *θ* = 0 or *θ* = 90° correspond to unidirectional laminates with fibers aligned or perpendicular to the specimen longitudinal axis, respectively.

Other building or lamination parameters were not changed, being not of interest for the current work. In practice, the default standard printing parameters of the machine were used.

**Figure 3:**
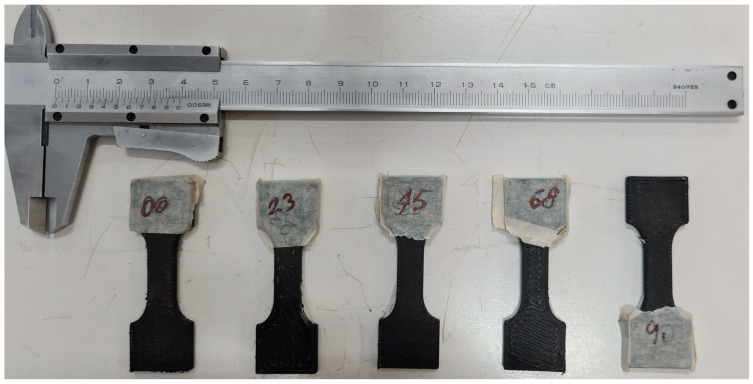
Example of some samples with the laminations described in Table 2, shown next to a Vernier caliper for comparison.

Figure 3 shows one sample for each lamination described in Table 2.

### 3.2. Experimental Results

Tensile tests according to ISO 527 were performed with a Zwick Z010 Pro Line universal material testing machine (ZwickRoell, Ulm, Germany) with simple wedge grips. The tests were conducted at a constant speed of 0.5 mm/s until rupture. Rupture was identified on the basis of a significant drop in the tensile load. Force–displacement curves were measured with a 10 kN load cell and a displacement transducer, encoder type, and recorded by means of the control unit of the machine and the testXpert™ III, version 1.11, testing software from Zwick. Data sampling is automatically managed by the testing software and is not particularly relevant for this low value of loading speed. The temperature was kept constant at room temperature.

**Figure 4:**
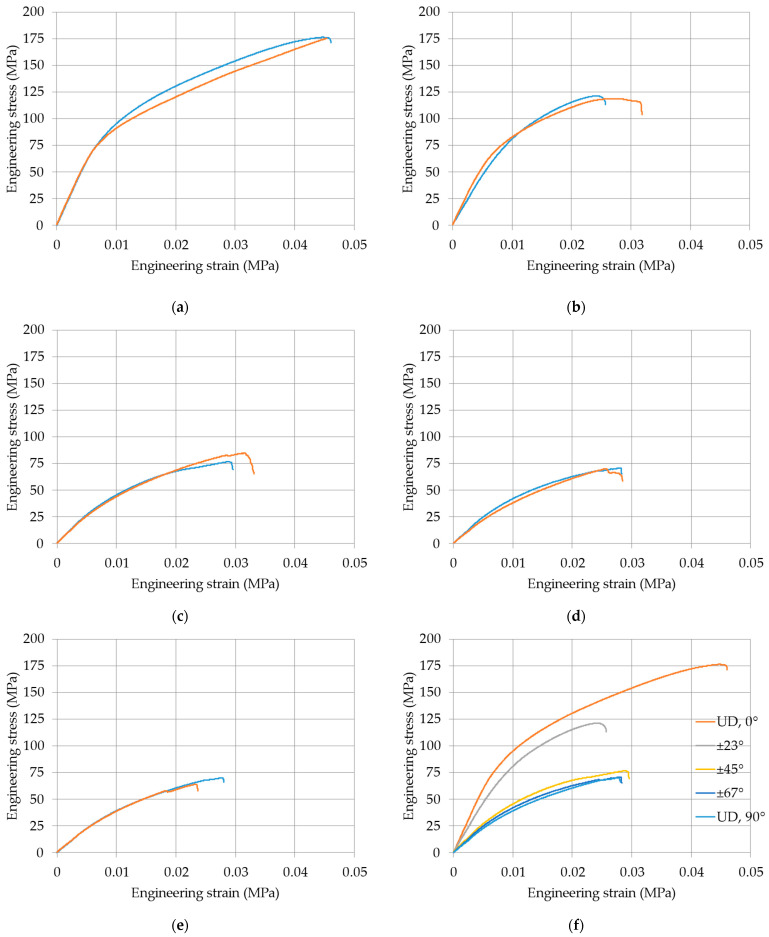
Typical stress–strain results from the tensile tests on the layered samples reinforced with glass fibers: (**a**) unidirectional 0°; (**b**) angle-ply ±23°; (**c**) angle-ply ±45°; (**d**) angle-ply ±67°; (**e**) unidirectional 0°; (**f**) comparison of the tested laminations.

In Figure 4, a curve for each lamination of the tested samples is reported.

From the experimental results, it appears that the tests were sufficiently repeatable (Figure 4a–e) in terms of maximum stress, strain, and overall stress–strain behavior. The variation in the peak load is within some percent, slightly more for the maximum strain as expected. In all cases, the maximum strain remains rather small, always less than 5%; the composite material remains sufficiently brittle. Failure is due to fiber fracture for the higher values of the fiber orientation when fibers are mostly loaded in tension and to matrix cracking and fiber–matrix debonding for smaller values of the fiber orientation when the matrix is mostly loaded in tension. In any case, it seems that failure criteria for composite materials are likely to be valid and applicable.

In general, the strength of the laminates decreases with the orientation of the fibers increasing from zero (Figure 4f), that is, less aligned with the longitudinal direction. The elongation at failure decreases as well, even if more scatter was measured, as is quite normal with this result. Table 3 reports the main results of the tests that will be used for the following analysis: the scatter in the two parameters is almost negligible for all cases, that is, all values of angles of fiber orientation.

**Figure 5:**
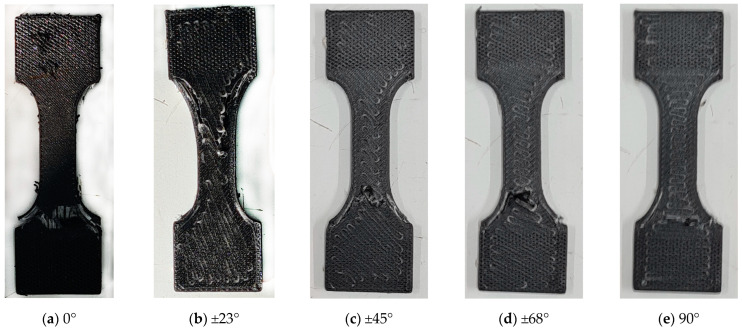
Samples reinforced with glass fibers after tensile tests: (**a**) unidirectional 0°; (**b**) angle-ply ±23°; (**c**) angle-ply ±45°; (**d**) angle-ply ±67°; (**e**) unidirectional 90°.

In Figure 5, some broken samples after the tensile tests are shown. The samples were not loaded until complete failure and separation; the tests were stopped after the failure occurred in one or some composite layers, and the load dropped by a significant amount. The information obtained was considered sufficient for the main aim of this work, which is to validate the model and to identify the relevant model parameters. Therefore, the type of failure is not really obvious, and it is presumably a combination of fiber failure and matrix cracking. This will be confirmed in the following analysis of the failure criteria.

### 3.3. Identification of the Model Parameters

#### 3.3.1. Evaluation of the Elastic Properties

**Figure 6:**
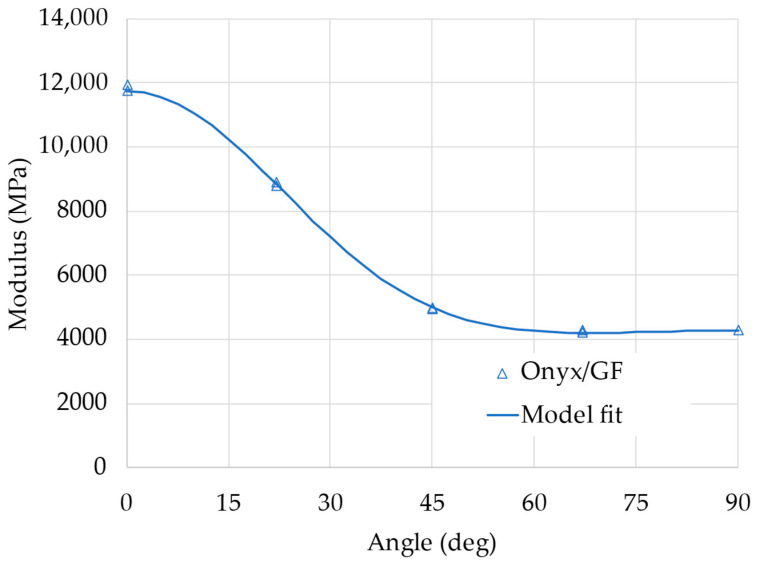
Experimental results of the tests on Onyx/GF samples in tension: analysis of the elastic properties.

The apparent elastic modulus of the samples with different fiber orientations depends on the elastic properties of the layers and on the angle, following the relations described by Equations (1)–(10). The layers’ elastic properties can then be estimated with a non-linear regression analysis by minimizing the square error between the experimental values of the apparent modulus and the value calculated by the model (from Equation (10)). Results from this analysis are reported in Figure 6; the curve represents the non-linear fit of the model, showing a very good correlation with the experimental data.

The identified elastic properties of the layers are reported in Table 4. It is important to compare the measured properties with the values of the elastic moduli of the Onyx and the glass fiber declared by the manufacturer (2.4 GPa and 21 GPa, respectively); the layers, made of a combination of the two materials, have elastic moduli along the two directions, which are between the elastic moduli of their components. This is an expected result, also justified by using classical micromechanical models for composite materials, such as, for example, the rule of mixture (RoM). It is not easy in this case to apply such micromechanical models, since the exact layer composition, that is, the fraction of matrix and fiber, is not known or easily evaluated. Moreover, models like the RoM only give a rough approximation of the moduli, which would not be particularly useful.

#### 3.3.2. Analysis of the Strength and Identification of a Suitable Failure Model

The strength of the tested samples has been studied to identify the strength properties of the layers with a similar non-linear regression analysis. For the strength, however, an additional problem regards the many applicable failure models, as described in Section 2.2. The models that have been applied to the experimental data are as follows:

- Tsai–Wu;
- Christensen;
- LaRC05;
- Hashin.

**Figure 7:**
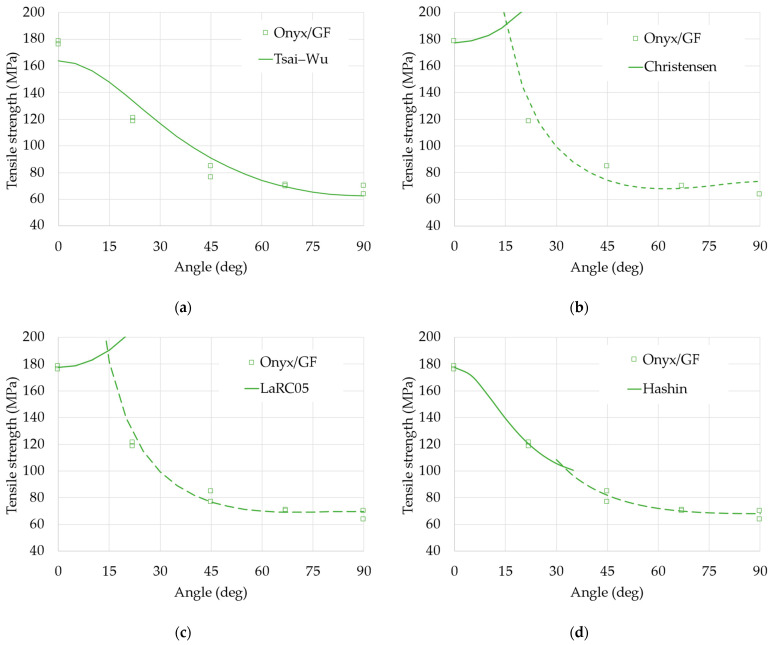
Experimental results of the tests on Onyx/GF samples in tension. Analysis of the strength and criteria: (**a**) Tsai–Wu model; (**b**) Christensen model; (**c**) LaRC05 model; (**d**) Hashin model.

The fit of the four models to the experimental data is shown in Figure 7. Note that the Christensen, LaRC05, and Hashin criteria distinguish between two different types of failure (fiber and matrix failure) and, therefore, two different inequalities (Equations (18), (21), and (23)). Therefore, the two inequalities can occur first depending on the combination of the stress components, and two curves represent the two possible failure modes.

The Tsai–Wu criterion shown in Figure 7a, although relatively simple, seems to give a sufficient approximation of the results, especially for the greatest values of orientation; that model does not distinguish between types of failure (it is described by only one continuous curve) and, therefore, is not able to fully describe the phenomena involved. On the other hand, the failure criteria from Christensen, shown in Figure 7b, and the LaRC05 model, shown in Figure 7c, are also not completely satisfactory even if both criteria distinguish between two types of failure, namely failure of the fibers (continuous lines) and failure of the matrix (dashed lines). The better fit, in this case, seems the Hashin criterion (the squared error is largely the minimum for this model). The Hashin criterion too, shown in Figure 7d, distinguishes between the two types of failure (fiber failure, continuous line, and matrix failure, dashed line) but uses a completely different formulation, energetically based for both cases, that apparently better represents the combination of failure modes observed from the samples tested; for smaller values of the orientation angle, the fibers are carrying the load and failure occurs in the fibers; for larger angles, the matrix fails first, lacking the contribution of the fibers’ strength.

The identified values of the strength parameters are reported in Table 5. If compared to the strength of the Onyx matrix and glass fiber (40 MPa and 590 MPa, respectively), the identified values seem sufficiently reasonable. Both the strength in the longitudinal and transverse directions of the layers lies between the values of the fiber and the matrix, as again it is predicted from the classical model as the RoM previously mentioned.

## 4. Conclusions

The mechanical behavior of a composite material obtained by an AM technique was studied in this work. Some papers have recently been published showing the applicability of the models used for classical composite materials to structures obtained by means of the FFF technique. However, most papers restrict the analysis to the stiffness and to simple lamination schemes that are not always accurate or complete for the analysis (few angles, asymmetric specimens, etc.).

In this paper, an innovative type of material for FFF was tested, considering the effect of different laminations, in particular showing the influence of the fiber orientation. To this aim, specimens of polyamide (Onix) reinforced with continuous glass fibers were analyzed. The experimental analysis was performed on symmetric angle-ply-layered samples. The experimental results showed the strong influence of the orientation angle, due to the high orthotropy of the base material.

The experimental results were then analyzed using the classical lamination theory to estimate the stiffness of the composite material specimen and using several failure criteria to estimate the strength properties. The models fit with good accuracy the experimental results and allow for identifying the material properties of the layer. The orthotropic stiffness properties (longitudinal, transverse, and tangential moduli and Poisson’s coefficient) of the fiber-reinforced layers were identified with a correlation coefficient of 99%. Among the various failure criteria considered, all are sufficiently apt to describe the failure of the considered material; the Hashin criterion gives the better fit with very good accuracy for any orientation angle (the correlation coefficient is again 99% for the Hashin criterion, even if the worst case corresponding to the simpler Tsai–Wu criterion still gives a value of 94%).

The proposed method is promising as a design tool to estimate the mechanical properties of FFF materials for future applications when high stiffness and strength are required in AM components.

## Figures and Tables

**Table 1:** Main properties of the materials used.

Material	Onyx	Glass Fiber
Elastic modulus (GPa)	2.4	21
Yield strength (MPa)	40	-
Ultimate strength (MPa)	37	590
Deformation at failure (%)	25	3.8
Density	1.2	1.5

**Table 2:** Plan of the prepared samples.

Angle	Lamination
0°	0_8_
23°	[±23]_4s_
45°	[±45]_4s_
68°	[±68]_4s_
90°	90_8_

**Table 3:** Main results in terms of global, apparent, elastic moduli, and tensile strengths of the tested samples.

Angle	Modulus (MPa)	Strength (MPa)
0°	11842 ± 135	177 ± 2
22.5°	8847 ± 63	120 ± 2
45°	4978 ± 15	81 ± 6
67.5°	4254 ± 53	71 ± 1
90°	4249 ± 46	67 ± 4

**Table 4:** Evaluated elastic properties of the orthotropic layers.

Property	Value
Elastic modulus along the fiber direction, *E_xx_*	11.7 GPa
Transverse elastic modulus, *E_yy_*	4.3 GPa
Poisson’s ratio, *ν_yx_*	0.37
Elastic tangential modulus, *G_xy_*	1.43 GPa_8_

**Table 5:** Evaluated strength properties of the orthotropic layers (Hashin criterion).

Property	Value
Tensile strength in the fiber direction, *X_t_*	178 MPa
Tensile strength in the transverse direction, *Y_t_*	67.9 MPa
In-plane shear strength, *S*	51.1 MPa

## Data Availability

The original contributions presented in the study are included in the article, further inquiries can be directed to the corresponding author.
